# Effects of automation trust in drivers’ visual distraction during automation

**DOI:** 10.1371/journal.pone.0257201

**Published:** 2021-09-14

**Authors:** Yijing Zhang, Jinfei Ma, Chunyang Pan, Ruosong Chang

**Affiliations:** School of Psychology, Liaoning Normal University, Dalian, China; Tongii University, CHINA

## Abstract

With ongoing improvements in vehicle automation, research on automation trust has attracted considerable attention. In order to explore effects of automation trust on drivers’ visual distraction, we designed a three-factor 2 (trust type: high trust group, low trust group) × 2 (video entertainment: variety-show videos, news videos) × 3 (measurement stage: 1–3) experiment. 48 drivers were recruited in Dalian, China for the experiment. With a driving simulator, we used detection-response tasks (DRT) to measure each driver’s performance. Their eye movements were recorded, and automation-trust scale was used to divide participants into high trust group and low trust group. The results show that: (1) drivers in the high trust group has lower mental workload and paid more attention to visual non-driving-related tasks; (2) video entertainment also has an impact on distraction behavior, variety-show videos catch more attention than news videos. The findings of the present study indicate that drivers with high automation trust are more likely to be involved in non-driving-related visual tasks.

## Introduction

Most people have heard of automated vehicles and feel positively about them [1]. According to the questionnaire survey, nearly 70% respondents believe that automated vehicles will occupy at least 50% of market share in the automotive industry by around 2050 [2]. However, taking a cautious stand, some researchers believe that vehicle automation will remain at a low level for a long time [3, 4]. Additionally, a number of studies have been carried out on human-machine interaction in L2 automated driving [5, 6]. In L2 automated driving, the vehicle is responsible for most of the operations, while the driver needs to monitor the vehicle conditions and hazards on the road and should be ready to take over the vehicle at any time (SAE Level 2, SAE International, 2016). Nevertheless, drivers’ supervising performance is not satisfactory: they either respond slowly to emergencies [7, 8] or are attracted too much by non-driving-related tasks [9, 10].

Trust plays a critical role in human–machine interaction due to the rapid advancement of technology [11]. Studies have shown that inappropriate automation trust exerts significantly negative influence on drivers’ supervisory behaviors [12, 13]. Automation trust can be divided into three layers: dispositional trust, situational trust, and learned trust. Learned trust can be further divided into before and after interaction [12]. The first type of learned trust is initially learned before human-machine interaction (e.g., through the influence such as attitudes, reputation of system, understanding and so on). The other type of trust is dynamically acquired during human–machine interaction. That is, a driver’s automation trust may change during his interaction with the vehicle. Numerous studies have verified the existence of dynamically acquired trust [14, 15].

People who do not trust automated vehicles will not purchase automated vehicles [16, 17]. However, drivers who trust automated vehicles were more likely to be distracted, which greatly damaged drivers’ alertness and jeopardizing traffic safety [13, 18]. That may be caused by driver’s mental workload insufficiency, and there is a subjective tendency to seek additional stimulation [19]. Some researchers conducted a follow-up study to distinguish the interestingness of tasks and found that interesting materials were less focused on but faster recognized [20, 21]. Thus, when studying the behaviors that might distract drivers, we need to pay attention to different natures of the same types of task while emphasizing the influence of different types of non-driving-related tasks (visual and audial) on driving safety.

In summary, we propose the following hypothesis:

**Hypothesis 1**: **Drivers in high trust group have a lower mental workload, and paid more attention to visual non-driving-related tasks.**
**Hypothesis 2**: **Video entertainment has an impact on distraction behavior, and variety-show videos catch more attention than news videos.**

## Methods

### Participants

From January to March 2018, we recruited 49 drivers aged 20 to 35 with no experience of using automated vehicles (*M* = 24.83, *SD* = 2.81) and with a driving history of 1 to 10 years (*M* = 2.94, *SD* = 2.06). The 49 drivers were randomly assigned to two groups to watch videos: the variety-show video group and news video group.

One Participant with an eye movement data collection rate of less than 80% was excluded. The remaining 48 drivers were randomly distributed into two groups. All subjects were in good health, with normal hearing and uncorrected visual acuity. The drivers were financially rewarded for their participation (100¥).

This study was approved by the Ethics Committee of Liaoning Normal University and was performed in accordance with the approved guidelines and the Declaration of Helsinki. The individual in this manuscript has been given written informed consent to publish these case details.

### Equipment

#### Driving simulator

**Fig 1:**
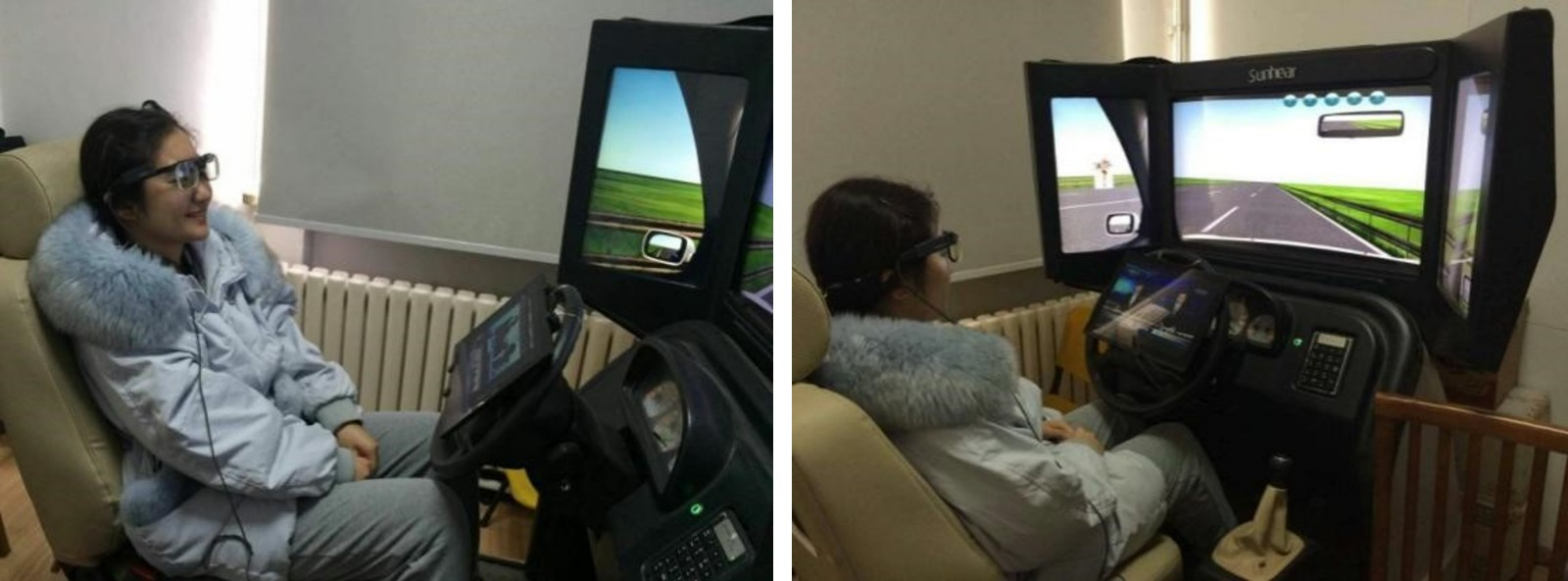
Automated driving scenario in the Sunheart QJ-3A1 Driving Simulator (small).

The Sunheart QJ-3A1 Driving Simulator (small) was used to simulate an automated driving scene on a monotonous highway without the driver’s steering, fueling. The DRT was randomly presented by the driving simulator on both sides of the screen, requiring the driver to brake the vehicle in response to pictures of pedestrians, and the task was presented repeatedly from every 20 s to 100 s, randomly, as shown in Fig 1(B).

#### Eye movement tracker

Eye-movement data were recorded using the head-mounted Tobii Pro Glasses II eye tracking system (Tobii, Sweden), which allowed free movement of the head. The sample frequency of the eye tracker was 50 Hz with an 82° × 52° recording visual angle.

#### Automation trust scale

The automation trust scale was compiled by Chien et al. [22]. This scale consists 21 questions, include items like: I can rely on automation to ensure my performance, Automation improves my performance, It is easy to follow what automation does… The answers to each question are divided into 5 levels from 1 to 5, with 1 meaning strongly disagree, and 5 meaning strongly agree. The participants gave their scores by their self-assessments.

#### NASA-Task load index

NASA-Task Load Index(NASA-TLX), developed by the National Aeronautics and Space Administration [23], has became an effective tool for subjectively assessing workload. This scale consists 6 dimensions, including mental demand, physical demand, effort, temporal demand, performance and frustration dimensions. Each dimension is divided into 10 levels from low to high.

#### Visual non-driving-related tasks

The visual non-driving-related tasks were presented on a tablet in the center of the steering wheel of the driving simulator. According to the interestingness of the videos, the non-driving-related tasks were divided into variety-show videos and news videos.

### Experimental design

A three-factor 2 (trust type: high trust group andlow trust group) × 2 (video type: variety-show videos news videos) × 3 (measurement stage: 1–3) experiment was designed for this study. automation trust and video entertainment were the between-subject variables, and driving stage was the within-subject variable.

#### Independent variables

The average score of drivers’ automation trust was measured as 3.331. The 24 drivers scored below that number were defined as the low trust group, and the rest were defined as the high trust group. The difference in the average scores of automation trust between the 2 groups reached statistical significance.

*Video entertainment*. To ensure significant differences between variety-show videos and news videos in terms of interestingness, 10 people were randomly selected to rate the interestingness of the videos on a scale from 1 to 10 (pre-experiment). According to the independent sample *t-test* results, there was a significant difference in interestingness between the variety-show videos and news videos (*t =* 3.130, *p<* .05). Hence, based on the subjective assessment of drivers, variety-show videos were significantly more interesting than news videos.

*Stage definition*. In order to avoid the influence of fatigue on the experimental results, we set the experimental duration to 30min [24–26], and each 10 min defined as a measurement stage.

#### Dependent variables

*Reaction time of DRT*. The driving simulator recorded the reaction times that the drivers needed to hit the brake pedal after the stimuli were presented.

*DRT accuracy*. If the driver responded within 2.1 s after the stimuli appeared, the DRT would be recorded as correct, and if the driver was unresponsive after 2.1 s, the DRT would be denoted as incorrect. 2.1 s was selected as the critical value because Gold et al. [21] found that 2.1s is the time that drivers needed to operate the vehicles once they noticed the takeover request.

*Total fixation duration*. This eye movement indicator represents the sum of duration of all fixation points in a specific area of interest.

**Fig 2:**
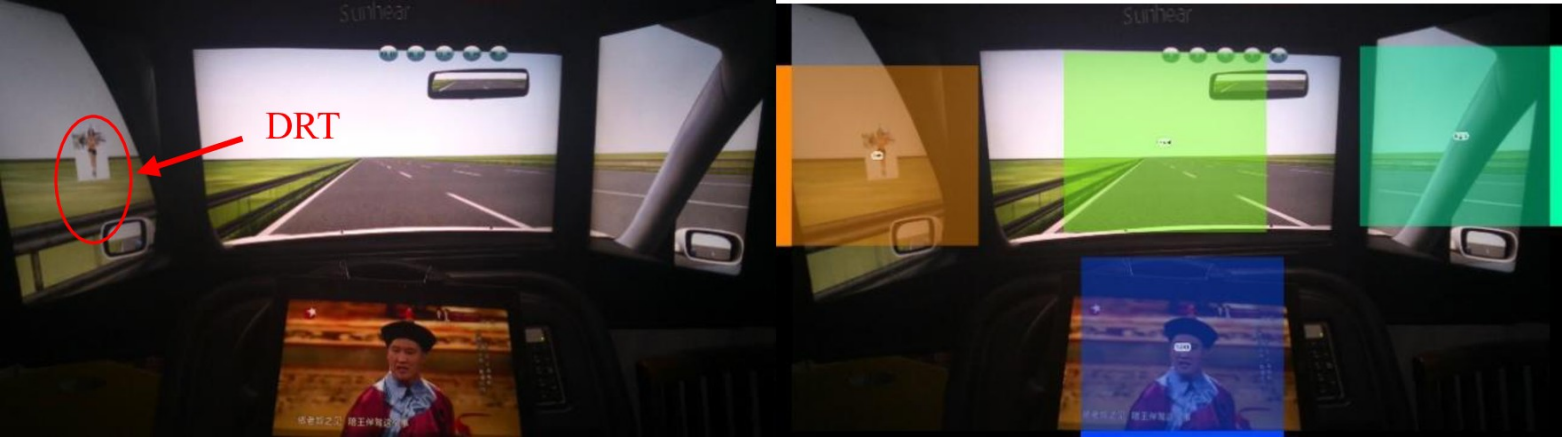
Division of area of interest of the portable eye tracker and collection of fixation duration indicators.

*Area of interests*. From the first-person perspective of the driver, the space in front of the driver was divided into 4 areas of equal size, namely the non-driving-related task area, the front area, the left-side area, and the right-side area (Fig 2).

*Subjective rating of automation trust*. Relevant scales were filled out by drivers before (pre-measurement) and after (post-measurement) the experiment.

*Subjective rating of the TLX*. The TLX was filled out by the drivers after the experiment.

### Procedure

1. Participants are asked to fill out the driver information form: including demographic information such as name, gender, age, driving age, and/or experience in using autonomous vehicles.
2. Let the Participants fill out the automation trust scale.
3. Inform the participants of the instruction, so that the participants understand that they need to hit the brake as soon as they see the randomly appearing pedestrians.
4. Give participants 5 minutes to practice the operations of the driving simulator and make sure they fully understand the instructions.
5. Length of the formal experiment is 30 minutes, consisting three 10-minutes stages.
6. Fill out the automation trust scale and task load scale again after the experiment.
7. After the formal experiment is over, the participants will be financially rewarded, and they will be verbally asked about the problems and psychological conditions encountered in the simulated driving. Thank the subjects for participating.

## Results

### Automation trust scale

To verify the influence of simulated automated driving experience on drivers’ automation trust and to explore the effectiveness of high trust group, a repeated measures ANOVA was performed with high trust group as the between-subject variable, driver automation trust measured before and after the experiment as the within-subject variable, and the automation trust score as the dependent variable. The results showed that the main effects of the high trust group were significantly different [*F*(1, 46) = 16.094, *p<* .001, *η*_*p*_^2^ = 0.259]. There was a significant interaction between the trust type and the scores measured before and after the experiment [*F*(1, 46) = 5.564, *p* = .023, *η*_*p*_^2^ = 0.108].

**Fig 3:**
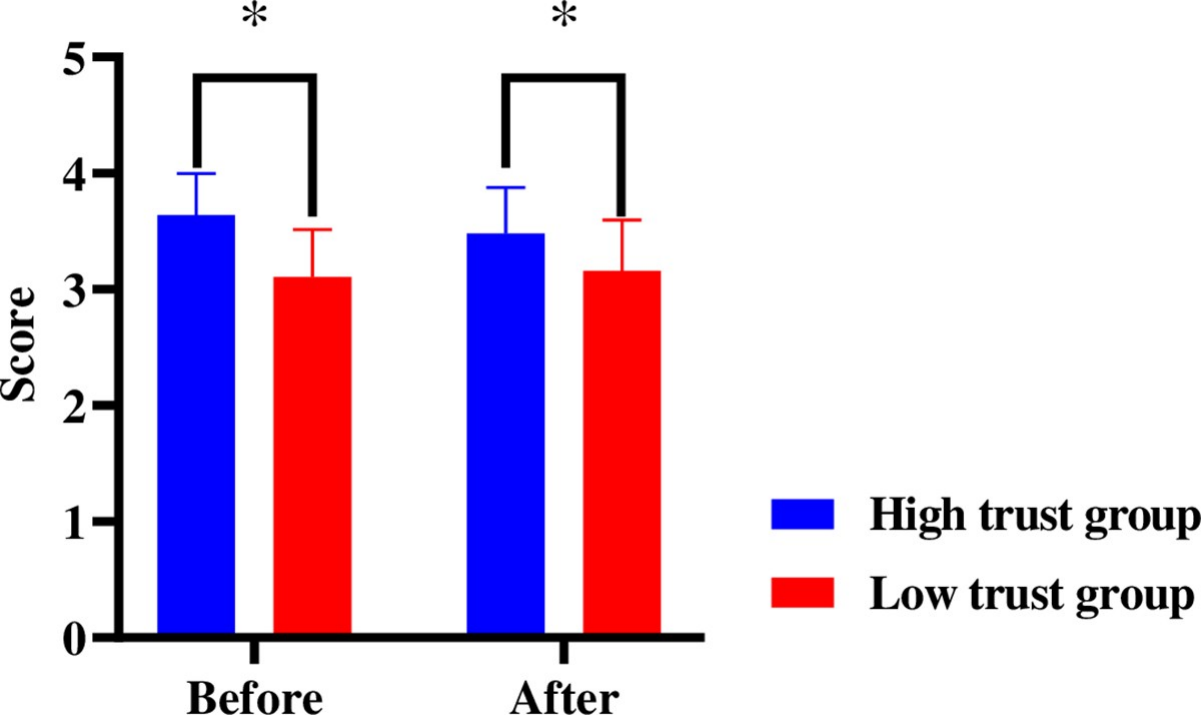
Measurement scores of drivers in the different trust groups. Note: *p< .05.

According to the simple effect-test, there were significant difference between drivers at different levels of trust toward automated driving before and after the measurement [*F*(1, 46) = 23.08, *p*< .001; *F* (1, 46) = 7.16, *p* = .010]. The scores of drivers in the high trust group were significantly higher than those in the low trust group both before and after the measurement, indicating that the drivers were correctly divided into high trust group and low trust group based on their real attitude (Fig 3).

### NASA-TLX

The independent sample *t*-test was performed on the scores obtained by drivers with different automation trust on the TLX. According to the results, there are significant differences between drivers with different automation trust on the 2 dimensions of the TLX. As well as the total score on the scale (Table 1).

**Table 1:** Analysis of mean of video type in different dimensions of the TLX scale (mean ± *SD*).

Dimension	high trust group	low trust	*t*	*p*
Cognitive load	3.83 *±* 1.76	5.58 *±* 2.125	-3.117	.003
Physical demand	2.92 *±* 1.64	2.96 *±* 1.68	-.087	.931
Effort level	3.96 ± 2.39	5.63 ± 2.41	-2.417	.020
Temporal demand	4.33 ± 2.46	4.00 ± 1.87	.529	.599
Frustration level	6.63 *±* 1.69	7.21 ± 1.44	-1.286	.205
Performance level	3.79 ± 1.96	3.92 ± 1.61	-.242	.810
Total score	4.24 ± 1.09	4.88 ± .91	-2.211	.032

There were significant differences between drivers in the high trust group and the low trust group in terms of the 2 dimensions of cognitive load and effort level (*t* = −3.11, *p* = .003; *t* = −2.41, *p* = .020), and the total score on the workload scale of drivers in the low trust group was significantly higher than that of drivers in the high trust group (*t* = −2.21, *p* = .032).

### DRT performance

#### DRT accuracy

With the trust type and video entertainment as the between-subject variables, the measurement stage as the within-subject variable, and the accuracy of the DRT as the dependent variable, a repeated measures ANOVA was performed. According to the results, the main effect of trust type was significant [*F*(1, 44) = 9.201, *p* = .004, *η*_*p*_^2^ = 0.173], as was the main effect of video entertainment [*F*(1, 44) = 6.582, *p* = .014, *η*_*p*_^2^ = 0.130]. There was a strong interaction among trust type, video entertainment, and measurement stage [*F*(2, 88) = 3.359, *p* = .039, *η*_*p*_^2^ = 0.071].

As indicated by the simple effect-test, in stage 1, the accuracy of different trust types varied greatly among the drivers watching variety-show videos [*F*(1, 45) = 13.41, *p* = .001]; the accuracy of drivers in the high trust group was significantly lower than that of drivers in the low trust group. The accuracy of the different trust types in the news video group was significant [*F*(1, 45) = 3.7, *p* = .041].

**Fig 4:**
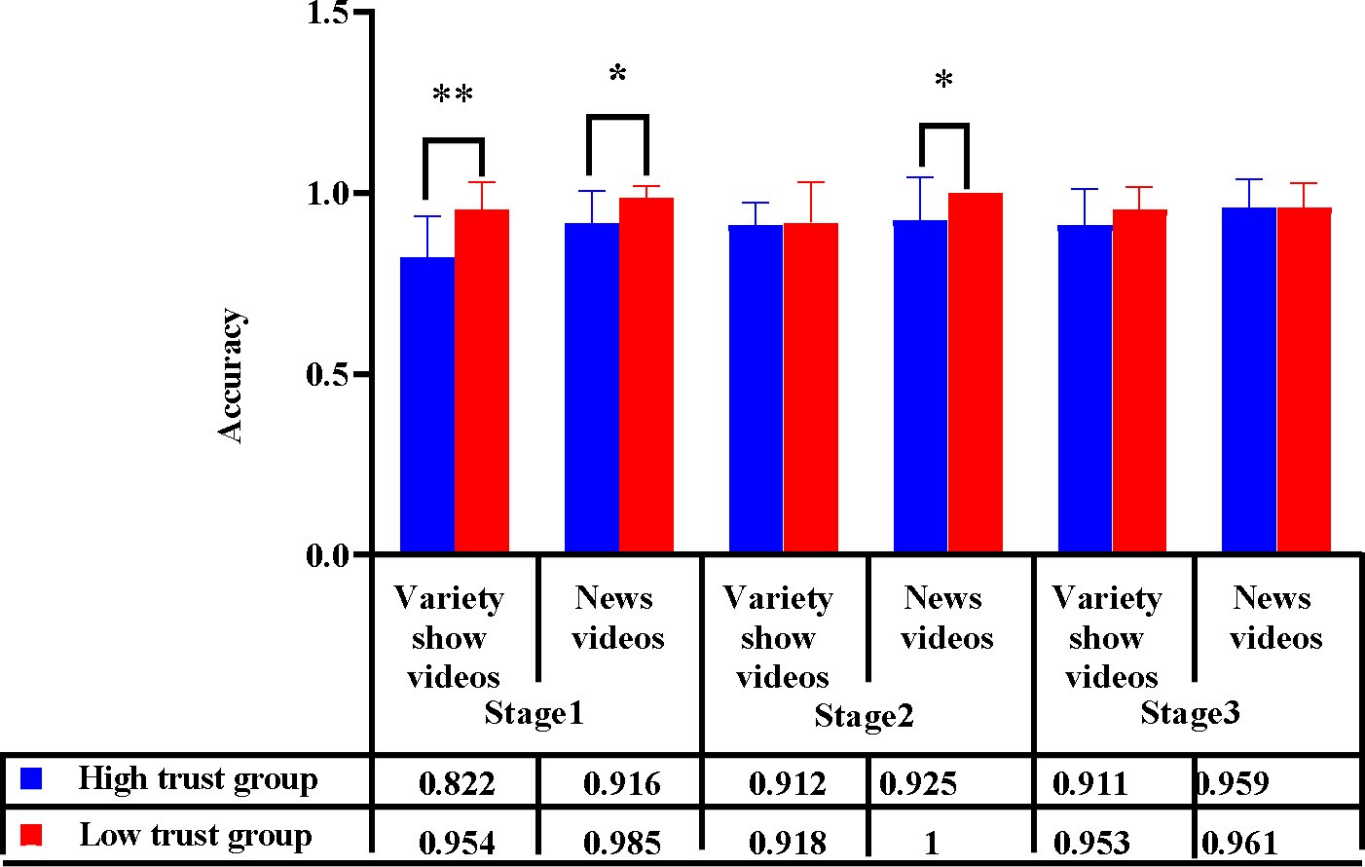
Influence of trust type and video entertainment on accuracy of takeover in 3 stages. Note:**p<0.05 *p< .05.

In stage 2, different trust types vary significantly when it comes to the accuracy of drivers watching news videos [*F*(1, 45) = 4.26, *p* = .045]; the accuracy of drivers in the high trust group is significantly lower than that of those distrusting automated driving. In stage 2, there was no difference between the automation trust of drivers watching variety-show videos. In stage 3, there was no apparent difference between the automation trust of drivers watching variety-show videos and news videos (Fig 4).

#### Reaction time of DRT

With the trust type and video entertainment as the between-subject variables, the measurement stage as the within-subject variable, and the reaction time of the DRT as the dependent variable, a repeated measures ANOVA was performed. The results indicated a significant main effect of the trust type [*F*(1, 44) = 6.216, *p* = .016, *η*_*p*_^2^ = 0.124] and video entertainment [*F*(1, 44) = 8.607, *p* = .007, *η*_*p*_^2^ = 0.155]. There was a significant interaction between trust type, video entertainment, and measurement stage [*F*(2, 88) = 3.851, *p* = .025, *η*_*p*_^2^ = 0.080].

**Fig 5:**
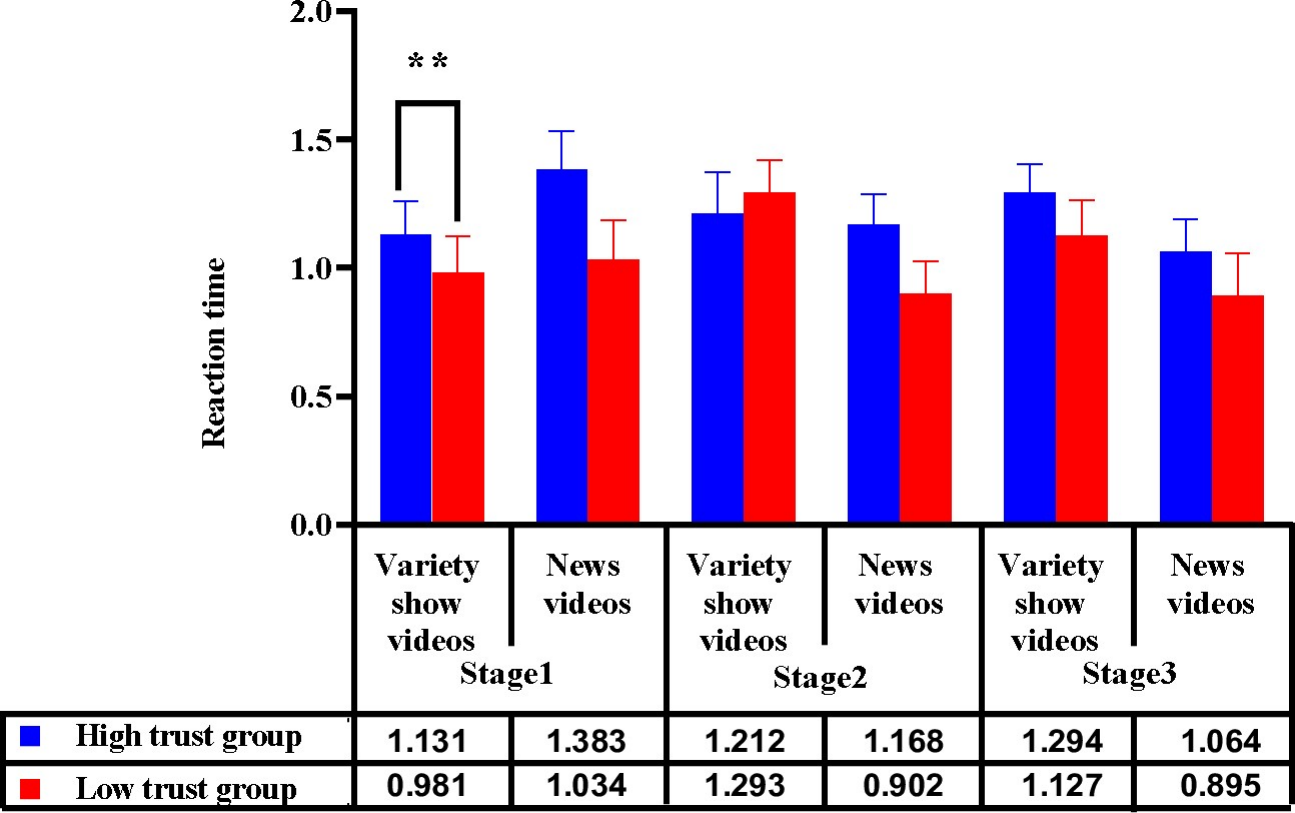
Influence of trust group and video entertainment on reaction time of takeover in 3 stages. Note: **p<0.05.

According to the simple effect-test, in stage 1, among the drivers watching variety-show videos, different automation trust types led to significantly different reaction times [*F*(1, 45) = 11.120, *p* = .002]; the reaction time of drivers in the low trust group was significantly shorter than that of drivers in the high trust group. In stage 2, among drivers watching news videos, there were insignificant differences between the reaction times of drivers and different automation trust types [*F*(1, 45) = 3.03, *p* = .089] (Fig 5).

### Eye movement indicators

#### Predictive effect of total fixation duration on DRT

Stepwise regression was carried out to analyze which variables had predictive effects on the overall accuracy of the 3 measurement stages. With the total accuracy of the 3 stages as the dependent variable, the age and gender of drivers were entered in the first step, and the total fixation duration in the front area, the non-driving related task area, and the left-side and right-side areas were entered in the second step. Table 2 shows the stepwise regression results.

**Table 2:** Hierarchical regression of gender and total fixation duration in the front area on accuracy.

Variable	*B*	*SE*	*β*	*R* ^2^	*R* ^2^_*adj*_	*F*
First step						
Constant	1.005	.031		.096	.076	4.801*
Gender	-.041	.019	-.310*			
Second step						
Constant	. 945	.031		.297	.265	9.312***
Gender	-.038	.017	-.286*			
Total fixation duration in the front area	6.252	.000	.449***			

Note:

*p< .05

***p< .001.

According to the results, the gender variable was able to explain 9.6% of the variation in the total accuracy [*R*^2^ = .096, *F*(1, 46) = 4.801, *p* = .034], which was then adjusted to 7.6%. The accuracy of female drivers was higher than that of male drivers. The total fixation duration in the front area could explain 29.7% of the variation in the total accuracy [*R*^2^ = .297, *F*(2, 46) = 9.312, *p*< .001], with the adjusted accuracy being 26.5%. The more the drivers fixed their attention in the front area during the driving process, the higher their accuracy were.

#### Fixation duration

**Fig 6:**
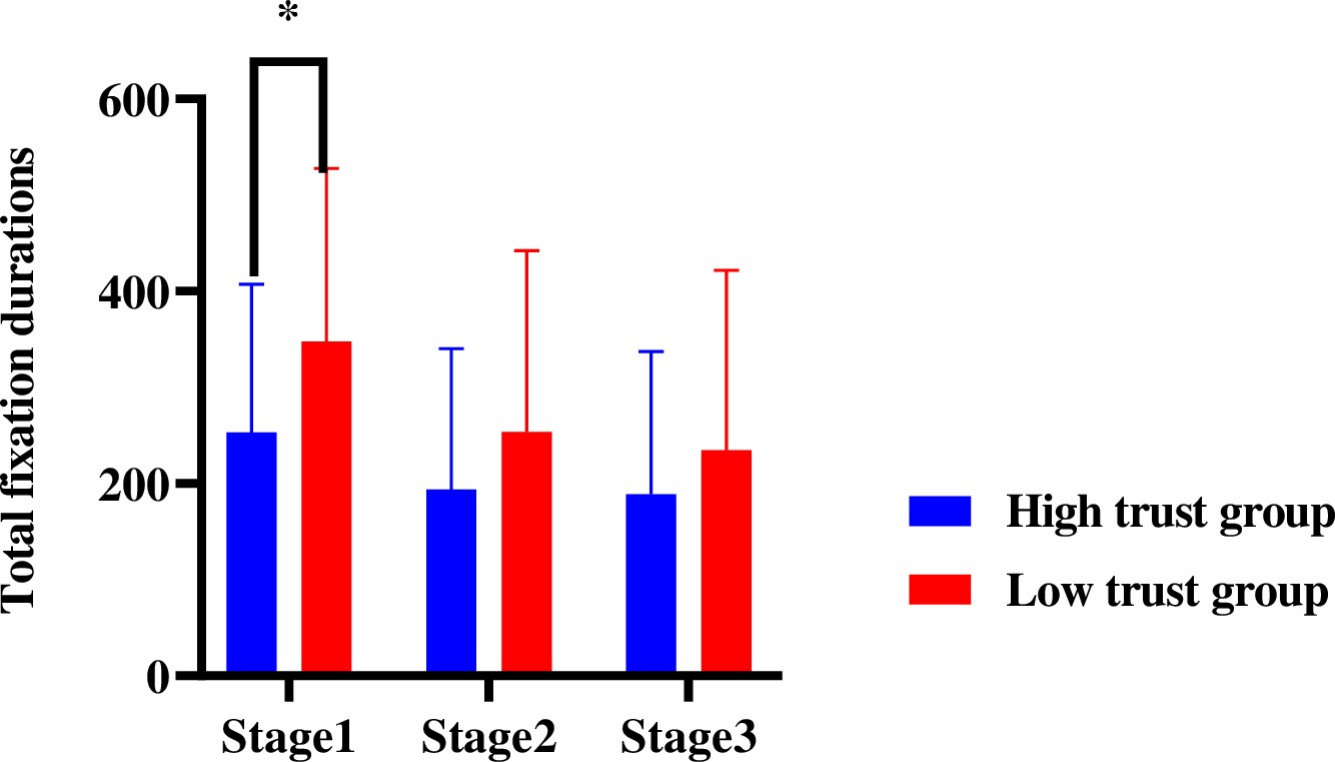
Total fixation durations in the front area of different automation trust during 3 stages. Note: **p<0.05.

With the trust type and video entertainment as the between-subject variables, the 3 measurement stages as the within-subject variables, and the total fixation duration in the front area as the dependent variable, a repeated measures ANOVA was conducted. According to the results, the main effect of the trust type was insignificant [*F*(1, 44) = 2.566, *p* = .117, *η*_*p*_^2^ = 0.056], while the main effect of video entertainment was significant [*F*(1, 44) = 7.641, p = .008, *η*_*p*_^2^ = 0.151], so was that of measurement stage [*F*(2, 88) = 7.641, p = .000, *η*_*p*_^2^ = 0.313] (Fig 6).

One-way ANOVA was performed with the trust type as the independent variable and the total fixation duration in the front area in stage 1 as the dependent variable. The results indicated a significant main effect of trust type [*F*(1, 47) = 3.711, *p* = .060, *η*_*p*_^2^ = 0.076]. In stage 1, the total fixation duration of drivers in the high trust group in the front area (253.402 *±* 153.713) was significantly shorter than that of drivers in the low trust group (347.653 *±* 180.031). However, when the data of stages 2 and 3 were included, the main effect of the trust type became insignificant (Fig 6).

**Fig 7:**
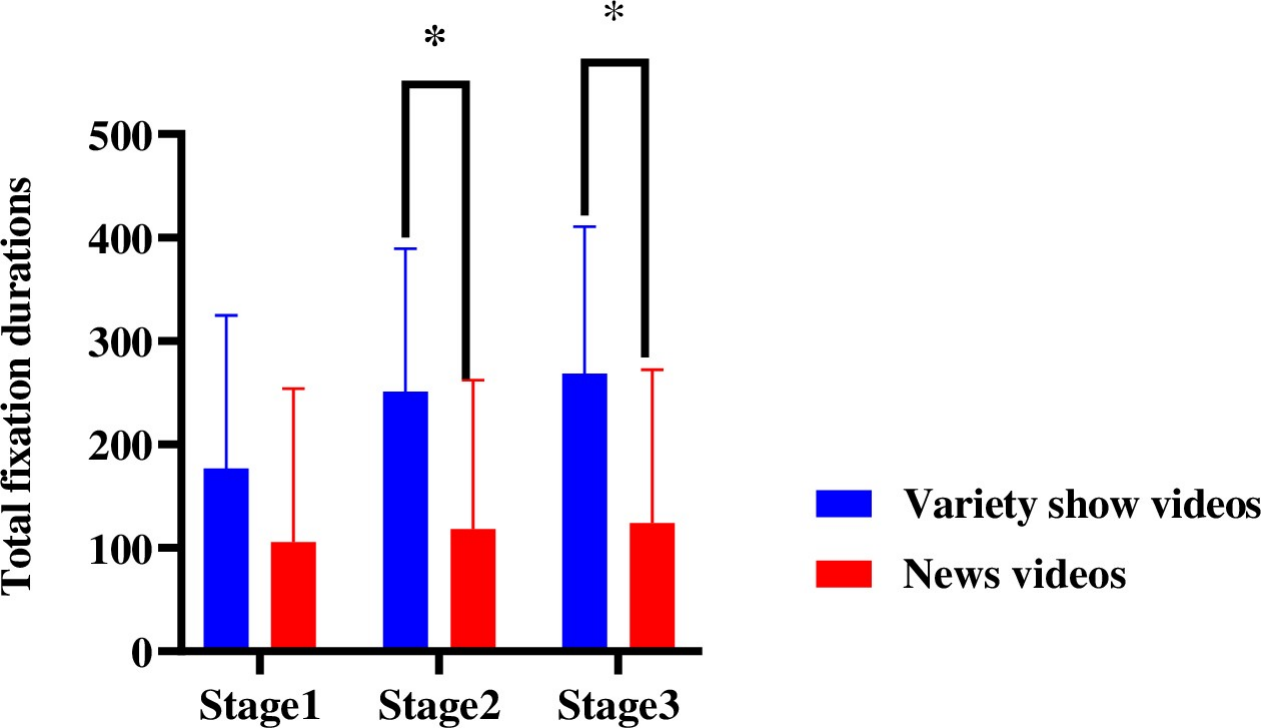
Total fixation durations in the NDRT area of different automation trust during 3 stages. Note: **p<0.05.

With the trust type and video entertainment as the between-subject variables, the 3 measurement stages as the within-subject variables, and the total fixation duration in the NDRT area as the dependent variable, a repeated measures ANOVA was conducted. According to the results, the main effect of the trust type was significant [*F*(1, 44) = 5.616, *p* = 0.022, *η*_*p*_^2^ = 0.116], while the main effect of video entertainment was significant [*F*(1, 44) = 10.617, p = 0.022, *η*_*p*_^2^ = 0.116], as was that of measurement stage [*F*(2, 88) = 9.987, p = 0.003, *η*_*p*_^2^ = 0.188].There was a strong interaction among video entertainment and measurement stages [*F*(2, 74) = 3.889, *p* = .025, *η*_*p*_^2^ = 0.095] (Fig 7).

#### Pupil diameter

With the trust type and video entertainment as the between-subject variables, the measurement stage as the within-subject variable, and the pupil diameter as the dependent variable, a repeated measures ANOVA was performed. According to the results, the main effect of trust type was insignificant [*F*(1, 37) = 1.269, *p* = .287, *η*_*p*_^2^ = 0.033], as was the main effect of video entertainment [*F*(1, 37) = 1.351, *p* = .253, *η*_*p*_^2^ = 0.035]. There was a strong interaction among trust type and measurement stage [*F*(2, 74) = 3.889, *p* = .025, *η*_*p*_^2^ = 0.095].

**Fig 8:**
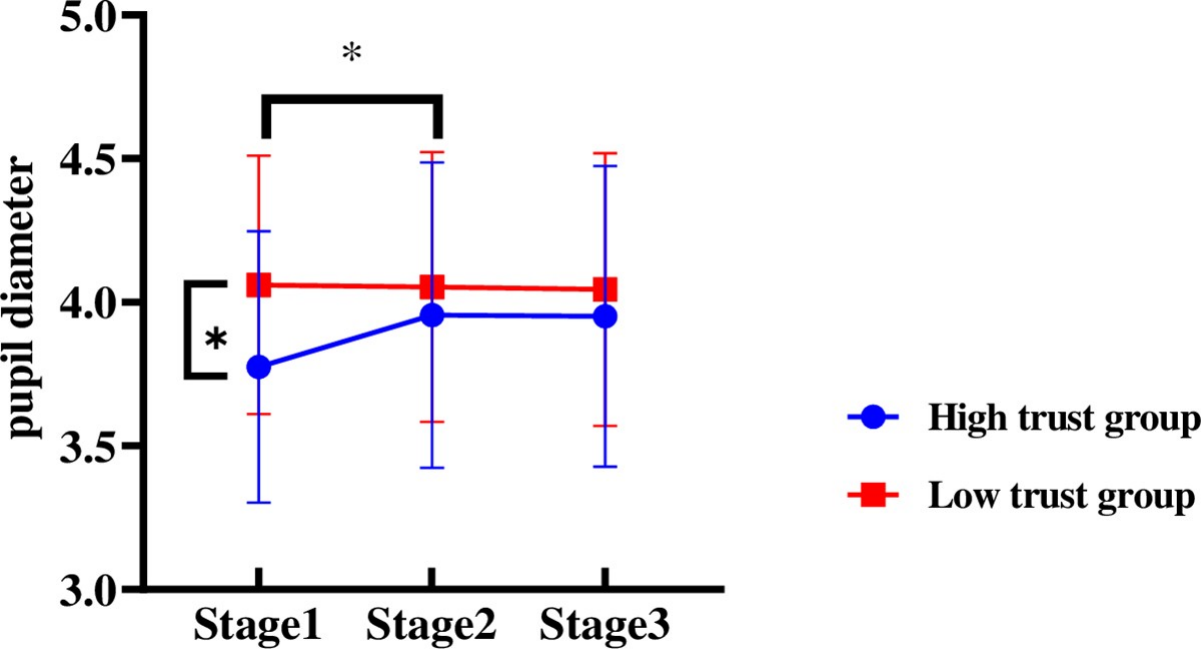
Driver’s pupil diameter of different automation trust during 3 stages. Note: **p<0.05.

As indicated by the simple effect-test, in stage 1, different trust types vary significantly in pupil diameter [*F*(1, 37) = 0.536, *p* = .0038]; The pupil diameter of drivers in the high trust group was significantly shorter than that of those in low trust group (Fig 8). In stage 2 and stage 3, the difference between drivers at different levels of automation trust is insignificant.

**Fig 9:**
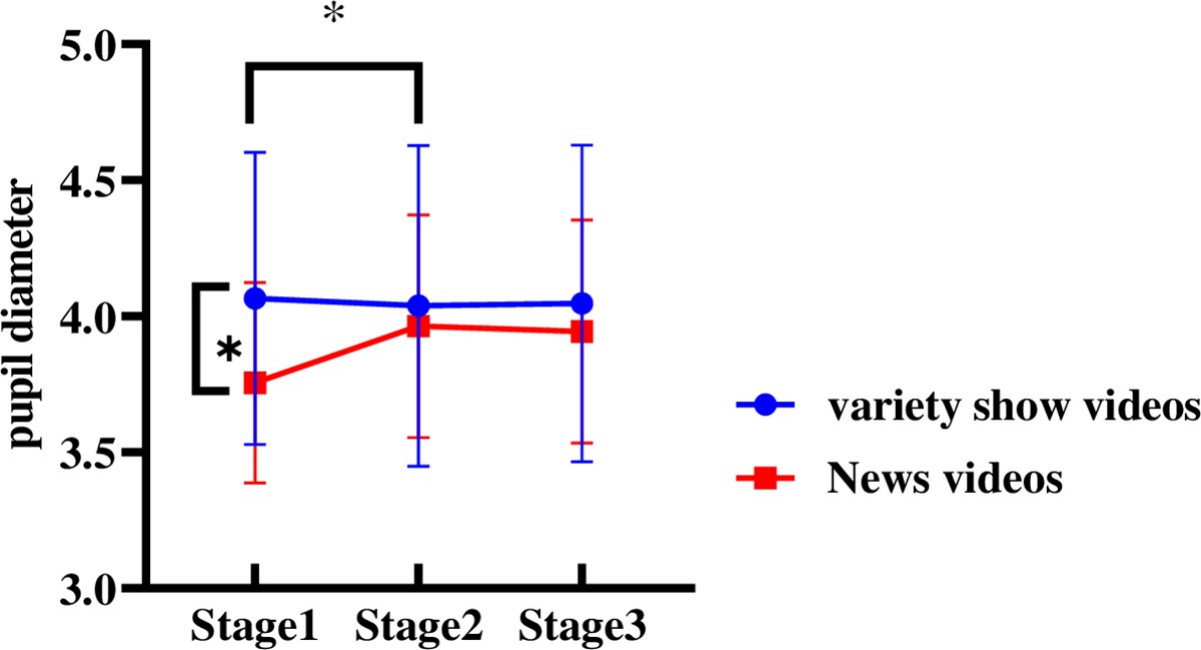
Driver’s pupil diameter of different video entertainment during 3 stages. Note: **p<0.05.

There was a significant interaction between video entertainment and measurement stage [*F*(2, 74) = 5.731, *p* = .007, *η*_*p*_^2^ = 0.127].As indicated by the simple effect-test, in stage 1, different video entertainment vary significantly in pupil diameter [*F*(1, 37) = 5.511, *p* = .0024]; The pupil diameter of drivers watching variety-show videos is significantly higher than that of those watching news videos (Fig 9). In stage 2 and stage 3, the pupil diameter of different types of trust was insignificant.

## Discussion

Based on drivers’ subjective assessments of workload, drivers in the high trust group showed lower cognitive load and effort levels than those in the low trust group, and the total score on the workload scale differed significantly between the 2 groups of drivers, which indicates that automation trust exerts a significant influence on driver mental workload. The pupil diameter of the eye movement data also supported this point. In the first stage, the pupil diameter of the low trust group was significantly higher than that of the high trust group. Previous studies have shown that the higher the mental workload, the larger the pupil diameter of the subjects would be [27, 28]. Seemingly more nervous, drivers in the low trust group more frequently scanned the driving-related areas, resulting in a high mental workload. However, drivers in the high trust group enjoyed the comfort of non-driving-related tasks in a more relaxed manner. They spent more time watching non-driving-related videos.

Drivers visually perceive most of the road conditions around them [29], and the occurrence of traffic accidents is significantly associated with excessively long visual distraction [30]. The longer the drivers do not focus on the road, the worse their lane-keeping ability appears to be [31]. This study not only confirmed this, but also further determined that the total fixation duration in the front area had a significant effect on predicting the DRT performance of drivers, yet it was unable to predict the situation in the peripheral areas (area with dangerous stimulus) and the non-driving-related task areas. Driving habits are the primary reason for this phenomenon [32]. In traditional driving tasks, the more drivers look at the center of the road, the better their driving performance will be. After participating in the automated driving task, the drivers did not change their habit of looking at the center of the road to maintain decent driving performance. Therefore, in the future design of human–machine interaction panels for automated vehicles, drivers’ driving habits should be fully respected, and drivers should be able to see the displayed information right in front of them or when they raise their head, so as to improve the efficiency of context information communication.

The experiences of automatic driving systems in other modes of transportation tells us that the drivers’ place in car cannot be replaced at present, and the system can only be a better assist other than a substitute. In order to improve the safety of autonomous driving technology, social awareness must be considered [33]. The results of this study fully show that the drivers’ inappropriate trust towards automated driving technology in certain situations will aggravate distracting behavior and is not conducive to traffic safety. Therefore, the development of social awareness algorithms for automated driving technology should take this into full consideration and include the drivers’ automation trust as variable.

## Limitations

This study has some limitations. Frequent DRT participation (20s~100s) required drivers to be highly alerted, which increased their mental workload, stress, and frustration [34]. In future studies, the influence of the frequency of DRT/take-over requests on drivers’ automation trust should be explored, and the experience can be divided into positive experiences and negative ones. In addition, though subjective rating is convenient and feasible, the results are not objective. Physiological measurements of driver mental workload are developing rapidly, and drivers’ eye-movements, heart-rate variability, and amplitude of brain waves can effectively reflect mental workload levels [35]. Future studies should improve the method for measuring mental workload and quantify the differences between driver mental workloads in different areas using objective indicators.

## Conclusion

Through the experiment of simulating autonomous vehicles with 48 drivers, we found that Drivers in the high trust group has lower mental workload and pay less attention to what is happening ahead, which leads to poor DRT performance.
